# An in-depth analysis of perinatal related mortality among women of South Asian ethnicity in Aotearoa New Zealand

**DOI:** 10.1186/s12884-023-05840-x

**Published:** 2023-07-24

**Authors:** Esti de Graaff, Lynn Sadler, Heena Lakhdhir, Rachel Simon-Kumar, Roshini Peiris-John, Wendy Burgess, Karaponi Okesene-Gafa, Robin Cronin, Lesley McCowan, Ngaire Anderson

**Affiliations:** 1 The University of Auckland Faculty of Medical and Health Sciences, Obstetrics & Gynaecology, Auckland, New Zealand; 2 Te Toka Tumai Auckland, Te Whatu Ora - Health New Zealand, Auckland, New Zealand; 3 Counties Manukau District, Division of Women’s Health, Te Whatu Ora - Health New Zealand, Auckland, New Zealand; 4 The University of Auckland School of Population Health, Auckland, New Zealand; 5 The University of Auckland Section of Epidemiology and Biostatistics, Auckland, New Zealand

**Keywords:** Stillbirth, Neonatal death, Perinatal mortality, Ethnicity, South Asia, Risk factor, Metabolic disorder, Gestational diabetes, New Zealand

## Abstract

**Background:**

International and national New Zealand (NZ) research has identified women of South Asian ethnicity at increased risk of perinatal mortality, in particular stillbirth, with calls for increased perinatal research among this ethnic group. We aimed to analyse differences in pregnancy outcomes and associated risk factors between South Asian, Māori, Pacific and NZ European women in Aotearoa NZ, with a focus on women of South Asian ethnicity, to ultimately understand the distinctive pathways leading to adverse events.

**Methods:**

Clinical data from perinatal deaths between 2008 and 2017 were provided by the NZ Perinatal and Maternal Mortality Review Committee, while national maternity and neonatal data, and singleton birth records from the same decade, were linked using the Statistics NZ Integrated Data Infrastructure for all births. Pregnancy outcomes and risk factors for stillbirth and neonatal death were compared between ethnicities with adjustment for pre-specified risk factors.

**Results:**

Women of South Asian ethnicity were at increased risk of stillbirth (aOR 1.51, 95%CI 1.29–1.77), and neonatal death (aOR 1.51, 95%CI 1.17–1.92), compared with NZ European. The highest perinatal related mortality rates among South Asian women were between 20–23 weeks gestation (between 0.8 and 1.3/1,000 ongoing pregnancies; *p* < 0.01 compared with NZ European) and at term, although differences by ethnicity at term were not apparent until ≥ 41 weeks (*p* < 0.01). No major differences in commonly described risk factors for stillbirth and neonatal death were observed between ethnicities. Among perinatal deaths, South Asian women were overrepresented in a range of metabolic-related disorders, such as gestational diabetes, pre-existing thyroid disease, or maternal red blood cell disorders (all *p* < 0.05 compared with NZ European).

**Conclusions:**

Consistent with previous reports, women of South Asian ethnicity in Aotearoa NZ were at increased risk of stillbirth and neonatal death compared with NZ European women, although only at extremely preterm (< 24 weeks) and post-term (≥ 41 weeks) gestations. While there were no major differences in established risk factors for stillbirth and neonatal death by ethnicity, metabolic-related factors were more common among South Asian women, which may contribute to adverse pregnancy outcomes in this ethnic group.

**Supplementary Information:**

The online version contains supplementary material available at 10.1186/s12884-023-05840-x.

## Background

Women of South Asian ethnicity have high rates of various pregnancy complications, both in South Asia and high-income countries. While India accounts for 17.7% of the global population and 17.3% of births worldwide (2020) [1], it carried 33.1% of the global stillbirth burden in 2019, with an estimated stillbirth rate of 18.2/1,000 total births (90%CI 17.6–22.1) [2]. India additionally ranked first in preterm births in 2010 (as an important risk factor for neonatal death [NND] [3], 23.6% of worldwide total), with an estimated mean preterm birth rate of 13.3% (95%CI 10.1–16.8) in South Asia [4].

Increased rates of stillbirth and perinatal mortality among South Asian women have long been reported in Australia [5], Aotearoa New Zealand (NZ) [6], the United Kingdom [7], Canada [8], and the Netherlands [9], with some British reports of ethnic disparities in pregnancy outcomes dating back to the 1980s [10, 11]. In particular, women of South Asian ethnicity have repeatedly been identified as having an independently increased risk for stillbirth [12–14], recently confirmed in a meta-analysis [15], while the association between South Asian ethnicity and NND is less clear [9, 16]. High rates of preterm birth among South Asian women have also been described [17, 18], however, the causal pathways leading to adverse pregnancy outcomes in these women remain largely unknown. In Aotearoa NZ the perinatal related mortality rate (PRMR; including deaths from 20 weeks gestation up to the 28^th^ day after birth) between 2008 and 2017 was highest for women of Indian ethnicity, with a rate of 15.4 per 1,000 total births [19]. In comparison, women of Māori, Pacific and New Zealand European (NZE) ethnicity had PRMRs of 10.7, 12.8, and 10.4 per 1,000 total births, respectively [19].

Australian studies have focussed on the increased rates of term stillbirths among women of South Asian ethnicity [20, 21], and iatrogenic birth or increased fetal monitoring ≤40 weeks gestation has been suggested as a potential intervention [22, 23]. While induction of labour at term may prevent some perinatal deaths [24, 25], it does not come without risk, as studies indicate increased neonatal morbidity [26, 27]. Women of South Asian ethnicity have a different pregnancy risk-profile [28], and differing gestation-specific placental pathology compared with women of other ethnicities [29, 30]. It is therefore important to understand the underlying pathology associated with the higher rates of perinatal death in South Asian women.

This study focusses on outcomes among South Asian women in Aotearoa NZ, due to their high perinatal death burden. As most South Asian women are immigrants to NZ, these mothers may experience barriers to health information and care [31]. Māori mothers, as the Indigenous people of Aotearoa NZ, and women of Pacific ethnicity were included in this study, as these women experience systemic, institutional, and personal barriers to healthcare that can lead to pregnancy complications [32]. As the largest ethnicity with comparatively favourable pregnancy outcomes, NZE women were included as the referent group in this study. By comparing these ethnic groups in this study we aim to broadly investigate ethnicity as a surrogate for social determinants of health, such as diet, cultural practices, and other environmental factors. We hypothesise that there are distinct ethnic causal pathways leading to perinatal death, with a strong metabolic component among women of South Asian ethnicity. The primary aim of this study was therefore to analyse differences in perinatal mortality between South Asian women and other ethnic groups in NZ, while a secondary aim was to identify risk factors contributing to stillbirth and NND by ethnicity.

## Methods

The study was approved by the University of Auckland Human Participants Ethics Committee (reference number 024201). Two data sources were used to investigate stillbirth and neonatal death by ethnicity in NZ: the PMMRC and the Statistics NZ Integrated Data Infrastructure (IDI), depending on data availability and quality of each source. While the PMMRC holds the highest-quality data for all perinatal related mortalities, the IDI includes higher-quality data on other variables of interest; for example ethnicity and socio-economic status for all live births. The PMMRC dataset was therefore used for all analyses including perinatal deaths only, while the IDI was accessed for most analyses which included live births in the denominator. See Additional file 1 for a background on both datasets. Some results were suppressed due to low counts, or secondarily suppressed to prohibit re-calculation, following PMMRC and Statistics NZ guidelines [33].

Singleton births to women of South Asian, Māori, Pacific and NZE ethnicity for the ten years 2008 to 2017 were included in this study. Ethnicity was self-defined [34], and grouped based on previous analyses using the sole-combination output method [28, 34]. The sole-combination ethnicity output method allows for additional ethnic categories when people identify with more than one ethnic group. For example, if a person identifies as both Indian and European, they are classified as Indian-European, as opposed to prioritised categorisation by a single ethnicity (Indian) only. Following previous recommendations, South Asian ethnicity was defined as Indian, Fijian Indian, South African Indian, Sri Lankan, Pakistani and Bangladeshi, since women from these ethnic groups present with similar pregnancy risk-profiles [28]. Births to women who identified with more than one ethnic group (Indian-Māori, Indian-Pacific and Indian-NZE) were classified according to their non-Indian ethnicity [28]. Among women of Māori, Pacific and NZE ethnicity, only women who recorded a single ethnic group were included in this study, as women with mixed ethnicities have previously been found to have differing pregnancy risk-profiles compared with women recording a single ethnicity only [28].

### Perinatal and maternal mortality review committee data

The PMMRC dataset was used for all numerator analyses, and a merged PMMRC dataset with the national Maternity Collection (MAT; see Additional file 1) was used to produce gestation- and cause-specific PRMRs by ethnicity, as the requisite variables for these figures were either unavailable or of lower quality in the IDI. For this study, cause of death was based on the second version of the Perinatal Society of Australia and New Zealand Perinatal Death Classification [35], as deaths prior to 2018 were classified according to this edition. Pre-existing maternal red blood cell disorders were defined as anaemia (low haemoglobin not further specified) and/or thalassemia trait (of any type, as a risk factor for anaemia in pregnancy [36]), by combining these two variables from the PMMRC dataset. However, the data does not specify whether women with thalassemia trait were also anaemic, or developed anaemia during pregnancy. Previous infertility was defined as a reported history of experienced infertility for more than 12 months prior to the index pregnancy, regardless of having received fertility treatment. Deprivation index was based on the NZ Socioeconomic Deprivation Indices, which is a decile score based on area of domicile [37]. In this study, quintile one represents 20% of the population living in the least deprived areas, while quintile five represents 20% of the population living in the most deprived areas. Statistical analyses were performed using SAS 9.4. Maternal and neonatal demographics were analysed using ANOVA or Kruskal–Wallis tests for continuous variables, which were adjusted for multiple comparison in post hoc analyses following the Tukey or Dwass-Steel-Critchlow-Fligner procedure. Chi-square or Fisher’s Exact tests were performed on categorical data, adjusted for multiple comparison using the Stepdown Bonferroni method. Statistical significance was set at *p* < 0.05. Customised birthweight centiles were calculated with the Gestation Related Optimal Weight customised centile calculator for NZ (accounting for maternal height, weight, ethnicity, parity and infant sex) from the Gestation Network [38]. Gestation- and cause-specific PRMRs by ethnicity were calculated following PMMRC methodology [39], in which gestational age at birth and cause of death were sourced from PMMRC data for the numerator, while ethnicity was sourced from MAT for both the numerator and denominator.

### Integrated data infrastructure data

The Statistics NZ IDI was used to prevent numerator-denominator bias evident in the merged PMMRC/MAT dataset, as variables are of varying quality. Analyses were performed using SAS 8.3 Enterprise Guide. Similar methods as described under PMMRC analyses were used to describe maternal and infant demographics, and to calculate customised birthweight centiles. Pregnancy outcomes included stillbirth (any death prior to birth from 20 weeks gestation), neonatal mortality (death of a live born infant from 20 weeks gestation up to the 28^th^ day after birth), and perinatal related mortality (as described previously). To investigate the effect of different ethnicity data sources (Births, Deaths and Marriages [BDM], MAT, and Census data) on PRMRs, source-specific rates were calculated among cases which had ethnicity data available from all three sources. Ethnicity data collection methods per source have been described elsewhere [40]. Pregnancy outcome rates were furthermore compared by logistic regression methods. Odds ratio (OR) and adjusted odds ratio (aOR) estimates, controlling for pre-specified risk factors, and profile-likelihood 95% confidence intervals were computed. Adjusted analyses included maternal age, body mass index (BMI), smoking status, parity, diabetic or hypertensive disorders (both pre-existing and pregnancy induced) and socio-economic status. The variable infant sex was only included for adjusted models on NNDs, as this data was missing for 28.5% of stillbirths. Several models were tested to identify predictors with the largest effect on birth outcome (see Table 1 for a description of each model). Women of NZE ethnicity were the referent group in all analyses. No between-group analyses were performed.

**Table 1:** Pregnancy outcomes by ethnicity, among total births using IDI data

*N* =		South Asian 24,273	Māori 56,700	Pacific 29,949	New Zealand European 250,314
**Pregnancy outcome**					
Perinatal related mortality	Rate/1000 total births	12.0	10.9	10.2	8.0
	OR (95%CI)	1.51 (1.33–1.70)	1.37 (1.25–1.50)	1.28 (1.13–1.44)	Ref
	aOR (95%CI)^a^	1.63 (1.43–1.85)	1.20 (1.07–1.34)	1.17 (1.02–1.35)	Ref
Stillbirth	Rate/1000 total births	8.9	6.8	7.0	5.9
	OR (95%CI)	1.52 (1.31–1.75)	1.16 (1.04–1.30)	1.20 (1.03–1.38)	Ref
	aOR (95%CI)^a^	1.67 (1.44–1.94)	1.05 (0.92–1.21)	1.12 (0.95–1.32)	Ref
	aOR (95%CI)^b^	1.61 (1.39–1.87)	1.15 (1.00–1.31)	1.10 (0.93–1.29)	Ref
	aOR (95%CI)^c^	1.63 (1.40–1.88)	1.09 (0.95–1.25)	1.25 (1.06–1.46)	Ref
Neonatal death	Rate/1000 live births	3.1	4.1	3.2	2.2
	OR (95%CI)	1.45 (1.13–1.84)	1.92 (1.64–2.23)	1.51 (1.21–1.86)	Ref
	aOR (95%CI)^a^	1.51 (1.17–1.92)	1.57 (1.29–1.90)	1.29 (1.01–1.65)	Ref
	aOR (95%CI)^d^	1.46 (1.13–1.86)	1.65 (1.36–2.00)	1.45 (1.14–1.83)	Ref
	aOR (95%CI)^e^	1.40 (1.09–1.78)	1.80 (1.50–2.17)	1.42 (1.11–1.79)	Ref

Births to women registered with a district health board were excluded from this analysis

^a^Adjusted: maternal age, body mass index, smoking status, parity, diabetic and hypertensive disorders (both pre-existing and pregnancy induced), and socio-economic status

^b^Adjusted: maternal age, body mass index, parity, diabetic and hypertensive disorders (both pre-existing and pregnancy induced), and socio-economic status

^c^Adjusted: maternal age, smoking status, parity, diabetic and hypertensive disorders (both pre-existing and pregnancy induced), and socio-economic status

^d^Adjusted: maternal age, smoking status, parity, diabetic and hypertensive disorders (both pre-existing and pregnancy induced), infant sex, and socio-economic status

^e^Adjusted: maternal age, parity, diabetic and hypertensive disorders (both pre-existing and pregnancy induced), infant sex, and socio-economic status

In NZ in 2017, 92.2% of pregnant women were registered with a lead maternity carer, and of these women, 94.1% registered with a midwife, 5.6% with an obstetric specialist, and 0.2% with a general practitioner [18]. The quality of data collection differs between births registered with lead maternity carers, and births to women registered with a district health board (hospital providers of primary maternity care). As these differences in data quality introduces systematic bias in analyses, all women with a district health board were excluded from analyses that utilised BMI, smoking, parity, or trimester of booking, due to a large amount of missing data. If gestational diabetes (GDM) or hypertensive disorders of pregnancy were not identified, these were assumed ‘absent’.

Finally, analyses identifying markers of risk for stillbirth and NND were performed, stratified by ethnicity. GDM was removed from both models as there was a large amount of missing data due to the majority of perinatal deaths occurring before 28 weeks gestation, while GDM screening in NZ is usually performed between 24 and 28 weeks [41]. An additional sensitivity analysis was therefore performed including GDM, on stillbirths from 30 weeks gestation; as women at this stage of pregnancy should have undergone GDM assessment [41]. As before, infant sex was excluded from the stillbirth risk model due to missing data. Deprivation quintile two was used as the referent group in risk factor analyses for NNDs, as the number of women residing in quintile one were too few for accurate comparison.

## Results

The PMMRC dataset identified 4,225 singleton deaths between 2008 and 2017, of which 420, 944, 678, and 2,183 were to women of South Asian, Māori, Pacific and NZE ethnicity, respectively. The IDI dataset identified 413,010 singleton births during the same decade to women of South Asian (*N* = 31,434), Māori (*N* = 67,632), Pacific (*N* = 47,991) and NZE (*N* = 265,953) ethnicity.

Among all births in the IDI, ethnicity data was sourced from BDM for 76.2% of stillbirths, 88.0% of NNDs and 99.3% of live births. The remaining cases had ethnicity data provided by the 2013 Census (16.5% stillbirths, 7.6% NNDs and 0.5% live births) and MAT (7.4% stillbirths, 4.3% NNDs and 0.2% live births). Demographic data in the IDI was therefore missing for a large proportion of perinatal deaths, although this varied by maternal ethnicity. Among stillbirths, BDM data was missing for 25.7% of South Asian, 34.6% of Māori, 37.8% of Pacific and 15.7% of NZE mothers. For NNDs BDM data was missing for 8.1% of South Asian, 20.8% of Māori, 12.9% of Pacific and 6.1% of NZE women.

### Demographic characteristics

Maternal and infant demographics by ethnicity and birth outcome are shown in Additional file 2 (PMMRC data) and Additional file 3 (IDI data). Maternal demographics were relatively similar by birth outcome (stillbirth or NND) within the same ethnic group, however differences by ethnicity were observed, such as in age, BMI, parity and socio-economic background.

PMMRC data further revealed differences in pre-existing and pregnancy-induced metabolic disorders by ethnicity, among women with perinatal death. Women of Pacific ethnicity had the greatest prevalence of pre-existing diabetes, although GDM rates were highest for South Asian mothers who experienced a stillbirth (4.3%, versus 1.2–2.5% among other ethnicities) and NND (7.6%, versus 1.7–3.8%). Pre-existing thyroid disease was also more common in South Asian compared to other ethnic groups. Furthermore, 13.2% (*N* = 40) of South Asian mothers with a stillbirth also had a maternal red blood cell disorder. Of these, 31 cases were classified as anaemic, while 11 had thalassemia (i.e. two were identified with both). Prevalence of maternal red blood cell disorders was much lower for mothers of Māori (5.2%), Pacific (4.8%) and NZE (2.1%, *p* < 0.01) ethnicity. Lastly, South Asian women with stillbirth were most likely to report that they had experienced infertility prior to the index pregnancy (13.2%, compared to 8.2% for NZE; *p* < 0.01).

Although birthweight differed by ethnicity in both data sources, customised birthweight centiles for stillbirths and NNDs did not differ from NZE.

### PMMRC data

**Fig. 1:**
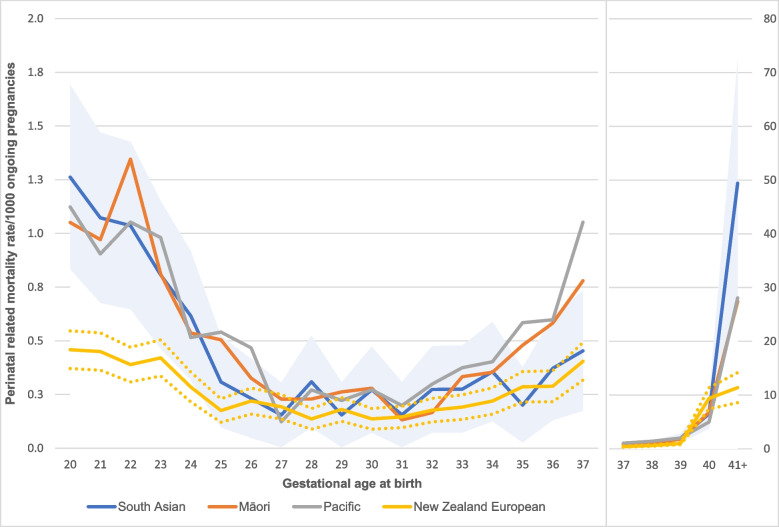
Gestational age-specific perinatal related mortality rates per 1,000 ongoing pregnancies, by ethnicity. 1) The blue shaded area indicates the 95% lower- and upper confidence interval (CI) for South Asian, while the yellow dashed lines indicate the 95% lower- and upper CI for New Zealand European; 2) Gestational age was sourced from the Perinatal and Maternal Mortality Review Committee dataset for the numerator, while it was sourced from the Maternity dataset for the denominator; 3) Ethnicity data was sourced from the Maternity dataset for both the numerator and denominator; 4) Terminations of pregnancy were excluded from this figure; 5) The scale of the secondary y-axis varies to allow for better visualisation of differences by ethnicity at term gestation

Gestational age specific PRMRs by ethnicity are shown in Fig. 1. See Additional file 4 for the table correlated to Fig. 1 (including rates by ethnicity and gestational age week). Overall, mortality rates were highest among extremely preterm infants (< 24 weeks gestation), and at term (> 37 weeks gestation). Women of NZE ethnicity had the lowest death rates across almost all gestations. At early gestations, PRMRs were highest between 20- and 22-weeks for South Asian mothers (1.3 and 1.1 per 1,000 ongoing pregnancies; *p* < 0.01 compared with NZE). In addition, all PRMRs ≤ 24 weeks gestation were significantly increased for South Asian, compared with NZE women (*p* < 0.05). A second rise in PRMRs was observed ≥ 41 weeks gestation, with a rate of 49.4 and 11.4/1,000 ongoing pregnancies for women of South Asian and NZE ethnicity (*p* < 0.01).

**Fig. 2:**
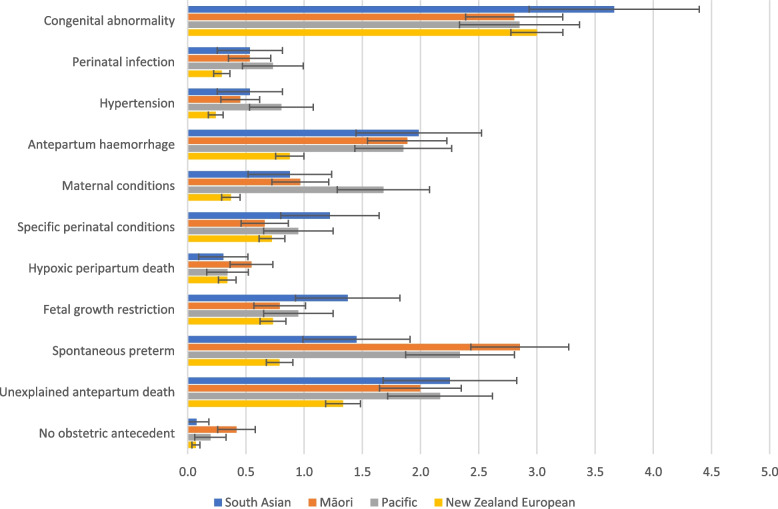
PSANZ cause of death-specific perinatal related mortality rates per 1,000 total births, by ethnicity. Ethnicity data were sourced from the Maternity Collection dataset for both numerator and denominator. The error bars indicate 95% confidence intervals

PRMRs by cause of death and ethnicity, are shown in Fig. 2. Congenital abnormality was most common among women of South Asian, Pacific and NZE ethnicity, while spontaneous preterm birth was the most frequent cause of death for Māori mothers (2.9/1,000 total births). Among South Asian and NZE women, the second highest cause-specific mortality rates were for unexplained stillbirth (2.3 and 1.3/1,000, respectively), while this was spontaneous preterm birth (2.3/1,000) for Pacific women, and congenital abnormality (2.8/1,000) for Māori women.

### IDI data

Rates and odds ratios of pregnancy outcomes by ethnicity are shown in Table 1. PRMRs (per 1,000 total births) were highest for South Asian women (12.0), followed by women of Māori (10.9), Pacific (10.2) and NZE (8.0) ethnicity. For South Asian, Pacific and NZE women, source-specific PRMRs did not differ significantly using BDM, MAT, or Census ethnicity data (Table 2). The largest difference was seen among Māori mothers, with a PRMR of 8.7/1,000 using BDM and Census ethnicity variables, and 7.7/1,000 with MAT data.

**Table 2:** Perinatal related mortality rates by ethnicity, using three different sources for ethnicity data

	**South Asian**	**Māori**	**Pacific**	**New Zealand European**
Births, Deaths and Marriages	10.59 (*N* = 21,816)	8.73 (*N* = 44,994)	8.51 (*N* = 32,079)	7.25 (*N* = 235,038)
2013 Census	10.58 (*N* = 21,555)	8.74 (*N* = 48,405)	8.42 (*N* = 32,064)	7.20 (*N* = 223,674)
Maternity Collection	10.67 (*N* = 17,715)	7.68 (*N* = 60,120)	8.23 (*N* = 37,173)	7.61 (*N* = 197,547)

Perinatal related mortality rates were only based on women with ethnicity data available from all three sources

South Asian women also had the highest stillbirth rates (8.9/1,000 total births), compared with Māori (6.8), Pacific (7.0) and NZE (5.9). Similarly, after adjustment, only women of South Asian ethnicity had a significantly higher risk of stillbirth, compared with NZE women (aOR 1.67, 95%CI 1.44–1.94). Further analyses revealed that removal of the variable for smoking from the multivariable model significantly increased the stillbirth odds for Māori mothers (aOR 1.15, 95%CI 1.00–1.31), while something similar occurred after removal of maternal BMI for Pacific mothers (aOR 1.25, 95%CI 1.06–1.46), illustrating the importance of these two confounding variables. In contrast, NND rates (per 1,000 live births) were highest for Māori women (4.1), followed by Pacific (3.2), South Asian (3.1), and NZE (2.2). A significantly higher adjusted odds was found across all ethnic groups, compared with NZE.

Risk factors associated with stillbirth and NND by ethnicity, are shown in Additional file 5. Advanced maternal age (≥ 35 years) and nulliparity were identified as a marker of risk for stillbirth, among all ethnic groups. Pre-existing diabetes (aOR1.85, 95%CI 1.01–3.40) and pre-existing hypertensive disorders (aOR 2.82, 95%CI 1.20–6.60) were risk factors for women of Pacific ethnicity only. Among NNDs, none of the variables were identified as risk factors across all ethnic groups. Multiparity was associated with a 50% reduced odds of NND for Pacific mothers (aOR 0.52, 95%CI 0.30–0.92), although this was not found in other ethnicities. Infant sex was not associated with NND in any ethnic group.

The results of the sensitivity analysis (including stillbirths ≥ 30 weeks gestation only, so that GDM could be included) are found in Additional file 6. Compared to the findings in Additional file 5, no large differences were observed, although low numbers prevented identification of several important risk factors. GDM was not identified as a marker of risk among NZE women, while results were suppressed due to low counts for mothers of other ethnic groups.

## Discussion

This study is the first to present a detailed review of perinatal related mortality by ethnicity and associated risk factors in NZ, using two of the highest quality national data sources. In line with previous research from high-income countries, our study identified women of South Asian ethnicity as having an independently increased chance of experiencing stillbirth, compared with NZE women [12–14]. Contrary to some studies [9, 16], South Asian mothers also had a 50% increased chance of experiencing a NND. Women of Māori and Pacific ethnicity had a higher odds of NND compared with NZE mothers following adjustment for confounders, but not for stillbirth. These findings are consistent with previous reports of privilege in health outcomes for European populations. Although the PRMR for South Asian women was consistent between ethnicity data sources, the PRMR for Māori women was 1.0/1,000 total births lower when ethnicity was sourced from the MAT dataset, rather than BDM or Census. Using MAT data alone would therefore underestimate the perinatal death burden among Māori mothers.

Consistent with Australian findings [20, 21], our data show a rise in perinatal death for women of South Asian ethnicity from 39 weeks gestation onward, similar to other ethnic groups. The PRMR then diverged from 41 weeks gestation and was fourfold higher compared with NZE women. However, these findings should be interpreted with caution, as South Asian women are overall more likely to be induced (31.9% across South Asian ethnicities) compared with women of other ethnic groups (between 20.3 and 25.8% for women of Māori, Pacific and NZE ethnicity) [28]. This is speculated to be related to clinical hesitancy towards expectant management for South Asian mothers past 40 weeks gestation, considering their higher rates of GDM, increased risk of abnormal post-term surveillance and need for obstetric intervention [42]. An artificial reduction of normal pregnancies in the denominator of this population may therefore have raised the ethnic-specific PRMR ≥ 41 weeks gestation, since PRMRs by ethnicity were not significantly different at 40 weeks. Alternatively, it may show that induction before 40 weeks gestation has artificially and successfully lowered PRMRs at those gestations [25]; however, this does not address the underlying cause of high PRMRs among South Asian women at term. Based on these analyses, we do not recommend iatrogenic delivery of infants to South Asian mothers at 40 weeks gestation. Moreover, although term stillbirths are often not inevitable and thus present an important group for stillbirth prevention [43], total numbers of term stillbirths were low compared with the absolute numbers of excess deaths among South Asian births at very preterm gestations. Our data suggests that focussing on research and interventions to lower extremely preterm deaths (< 24 weeks gestation), would result in a larger reduction in the death burden. This is consistent with findings from the 15^th^ annual NZ PMMRC report, where the second highest NND risk (per 1,000 ongoing pregnancies) for infants born between 20- and 24-weeks gestation, occurred amongst South Asian mothers [6].

This study furthermore suggests that aetiologies leading to perinatal death may differ by ethnicity. Although we were unable to adjust for these variables in analysis, women of South Asian ethnicity with perinatal death were overrepresented in several metabolic-related disorders, such as GDM, thyroid disease, red blood cell disorders and to a lesser extent pre-existing diabetes. Studies in Norway and the United Kingdom have additionally identified significantly increased rates of subclinical hypothyroidism among South Asian women, compared with other ethnic groups [44, 45]. These disorders may have underlying metabolic pathology in common, as previous research has found a link between (1) insulin resistance and thyroid dysfunction [46], (2) altered glucose metabolism, thalassemia and possibly iron deficiency [47, 48], and (3) iron deficiency anaemia and thyroid metabolism [49]. Another diagnosis associated with insulin resistance and more commonly diagnosed in South Asian women, is polycystic ovarian syndrome (PCOS) [50]. PCOS is additionally associated with infertility [51]. While our data could not identify women with PCOS in this study, we did find a significantly larger proportion of South Asian women with perinatal death who had previously experienced infertility. Although it would require appropriate investigation, we hypothesise that targeting metabolic-related disorders pre-conception may help prevent some of the excess pregnancy complications in South Asian women. Further research into social determinants of health associated with metabolic-related disorders, such as diet or environmental toxins, should be given consideration. Ultimately this may lead to evidence-based interventions focussed on prevention, rather than risk mitigation by earlier iatrogenic delivery. As social determinants may change over generations of migration, the effect on metabolic health and pregnancy outcome should be monitored.

Finally, risk factor analyses found that advanced maternal age (≥ 35 years) and nulliparity increased the stillbirth odds among all ethnic groups [52], with the strongest effect of advanced maternal age in South Asian women. South Asian mothers birthing in NZ are more often nulliparous and among the older mothers, leading to an increased burden [28]. Women of South Asian ethnicity could therefore be advised on the age related risks pre-conception, in order to make informed decisions around family planning. Maternal smoking and BMI also had a strong influence on stillbirth and NND odds (Table 1), specific to women of Māori and Pacific ethnicity. Although neither variable was identified as a marker of risk in stratified analyses for Māori and Pacific women (Additional file 5), both were significant for NZE. This is therefore likely to represent a power issue. Other previously documented risk factors for adverse pregnancy outcome were not confirmed in this study. For example, while an exponential relationship between socio-economic deprivation and perinatal mortality has been described both in NZ and internationally [19, 52], with the highest numbers of deaths occurring in mothers who reside in the most deprived areas, we were not able to confirm this. Secondly, despite the established association between male infant sex and NND [53, 54], this was not identified as a risk factor in any ethnic group. In contrast, both type 1 and type 2 diabetes were identified as risk factors for perinatal mortality, consistent with existing literature [55], but we were unable to identify a relationship between GDM and stillbirth [56].

### Strengths and limitations

A strength of this study was the combined use of the two highest quality data sources available in the NZ maternity setting (with regards to data accuracy), enabling us to perform detailed analyses by ethnicity. Both data sources provided us with the most reliable ethnicity data [34, 40]. This study is therefore the first to perform an in-depth analysis of perinatal mortality by ethnicity in NZ, using the highest quality ethnicity data in the numerator and denominator, with a specific focus on women of South Asian ethnicity.

There were also limitations to this study, some of which are specific to the IDI. For example, missing BDM data resulted in a large amount of missing demographic information for stillbirths. As discussed previously, we suspect a strong underreporting of GDM in the IDI data sources. Although missing GDM data limited our analyses, a lack of association with perinatal death in the subset ≥ 30 weeks is reassuring and consistent with international research. Rates of pre-existing diabetes may be similarly underestimated. In 2016 HbA1c at booking was introduced in NZ, which is likely to have increased detection of previously undiagnosed pre-existing diabetes, although this study only included deaths between 2008 and 2017. Additionally, women facing barriers to access care, including those living in more deprived areas, may not have performed diabetes testing. For example, a 2020 retrospective review of clinical records showed that screening rates for diabetes in pregnancy were significantly lower among Māori mothers (17.5%, compared with non-Māori 31.6%) [57]. Other metabolic disorders, such as thyroid disease, PCOS and red blood cell disorders, are not currently collected in national maternity datasets. We therefore recommend that national datasets should begin to universally collect these data, for improvement of future research on high-risk groups. Although we acknowledge that South Asian women in this study reported increased experienced infertility prior to the index pregnancy, this does not necessarily reflect the prevalence of PCOS and reporting of infertility may differ by ethnicity. These weaknesses limit analyses on important risk factors specific to women of South Asian ethnicity. Other strengths and limitations of perinatal research in the IDI have been extensively reported elsewhere [40].

## Conclusions

In conclusion, women of South Asian ethnicity in Aotearoa NZ were at independently increased risk of both stillbirth and NND compared with NZE women. Māori and Pacific mothers were at increased risk for NND, but not stillbirth. Risk factors for adverse outcome did not differ significantly by ethnicity in this study. However, amongst women who had a perinatal death, South Asian mothers had higher rates of metabolic-related disorders, which presents an interesting avenue for future research and possibly pre-conception or antenatal interventions. Furthermore, South Asian women had increased PRMRs both at extremely preterm gestation (≤ 24 weeks) and ≥ 41 weeks. It may be that the increased PRMRs among term deaths is confounded by a larger proportion of uncomplicated South Asian pregnancies undergoing induction of labour, and therefore caution is recommended with interpretation of this finding.

## Disclaimer

These results are not official statistics. They have been created for research purposes from the Integrated Data Infrastructure (IDI), which is carefully managed by Stats NZ. For more information about the IDI please visit https://www.stats.govt.nz/integrated-data/.

## Supplementary Information

**Additional file 1.** Background to the datasets in this study.

**Additional file 2.** Maternal and neonatal characteristics by ethnicity, among perinatal deaths using PMMRCdata.

**Additional file 3.** Maternal and neonatal characteristics by ethnicity, among all births using IDI data.

**Additional file 4.** Gestational age-specific perinatal related mortality rates per 1,000 ongoing pregnancies, by ethnicity.

**Additional file 5.** Risk factors for stillbirth and neonatal death by ethnicity, using IDI data.

**Additional file 6.** Risk factors for stillbirth from 30 weeks gestation by ethnicity, using IDI data.

## Data Availability

The data that support the findings of this study are available from the Perinatal and Maternal Mortality Review Committee and the Statistics New Zealand Integrated Data Infrastructure, but restriction apply to the availability of these data, which were used under license for the current study, and so are not publicly available. Data can only be made available with permission of the individual data custodians. Please contact the corresponding author (EdG) for any data requests.

## Abbreviations

BDM: Births, Deaths and Marriages
BMI: Body mass index
GDM: Gestational diabetes
IDI: Integrated Data Infrastructure
MAT: Maternity Collection
NND: Neonatal death
NZ: New Zealand
NZE: New Zealand European
PCOS: Polycystic ovarian syndrome
PMMRC: Perinatal and Maternal Mortality Review Committee
PRMR: Perinatal related mortality rate
