# Supplementary material for: An in-depth analysis of perinatal related mortality among women of South Asian ethnicity in Aotearoa New Zealand
Source: BMC Pregnancy Childbirth. 2023 Jul 24;23:535. doi: 10.1186/s12884-023-05840-x (PMC10364368; doi:10.1186/s12884-023-05840-x)
Supplement: Supplementary file 1 — Additional file 1. Background to the datasets in this study. [file 12884_2023_5840_MOESM1_ESM.pdf]

Additional file 1 - Background to the datasets in this study

Perinatal and Maternal Mortality Review Committee data

The highest quality perinatal mortality data is collected and held by the NZ Perinatal and Maternal Mortality Review Committee (PMMRC) [1]; an independent committee under the Health Quality & Safety Commission NZ, which is recognised internationally [2]. Deaths are notified within 48 hours of a perinatal death event by the lead maternity carer or another clinician, after which details of the deaths of stillborn fetuses and neonates (all perinatal related mortality from 20 weeks gestation up to the 28th day after birth) are collected by the PMMRC using a standardised reporting tool. This contains information on the mother (e.g. demographic, medical, and obstetric history), the infant, and details on the pregnancy and birth event. Each case is subsequently reviewed by a local multidisciplinary team, after which a perinatal death classification form is completed, and all data is submitted to the PMMRC. Data are validated regularly to eliminate duplicates, complete missing information, or rectify inconsistencies. In addition, a total perinatal dataset of all births is created by merging PMMRC data with the national Maternity Collection (MAT) from the Manatū Hauora (Ministry of Health) [3]. This merged dataset thus includes both perinatal deaths (numerator) and live births (denominator), with a 96% match between the deaths in PMMRC and their corresponding birth event in MAT. Mortality Review Committee data

Integrated Data Infrastructure data

The IDI is a collection of NZ government and non-government administrative and survey data, held by Statistics NZ, which was accessed to create a perinatal dataset for this study. A comprehensive methodology, including a detailed explanation of the used data sources, has been described elsewhere [1]. In short, national maternity and neonatal data of singleton births between 2008 and 2017 were linked, including all birth events from 20 weeks gestation. The two most important national datasets for perinatal research are MAT (clinical data on all birth events), and Births, Deaths and Marriages (BDM; infant and parent demographic data on all

deliveries). Both datasets have limitations specific to perinatal deaths, which may result in missing data including gestational age, birthweight and infant sex. This is because MAT only records infant demographics for live births, while BDM data can be incomplete for perinatal deaths due to reliance on parental completion of registration of the birth. Finally, individual ethnicity data for mothers in this study was collected from BDM, the 2013 Census and MAT datasets; and used in that order depending on data availability.

[1]. de Graaff EC, Sadler L, McCowan L, Cronin R, Anderson N. A comprehensive methodology report for maternity and perinatal mortality research in the New Zealand Integrated Data Infrastructure. N Z Med J. 2023;136(1569):37-49.

[2]. Helps Ä, Leitao S, Gutman A, Greene R, O'Donoghue K. National perinatal mortality audits and resultant initiatives in four countries. Eur J Obstet Gynecol Reprod Biol. 2021;267:111-9.

[3]. Perinatal and Maternal Mortality Review Committee. Methodology and definitions for Perinatal and Maternal Mortality Review Committee (PMMRC) reporting. Wellington: Health Quality & Safety Commission: Health Quality & Safety Commission; 2018.
